# Integrating community health workers in early childhood well-child care: a statement from the Pediatric Academic Societies Maternal Child Health: First 1,000 days Special Interest Group

**DOI:** 10.1186/s12875-024-02582-3

**Published:** 2024-09-27

**Authors:** Tumaini Rucker Coker, Emily F. Gregory, Mary McCord, Rushina Cholera, Hayes Bakken, Steve Chapman, Eimaan Anwar, Jennifer Lee, Shauntée Henry, Lisa J. Chamberlain

**Affiliations:** 1 University of Washington and Seattle Children’s, Seattle, WA USA; 2 Children’s Hospital of Philadelphia, Philadelphia, PA USA; 3 NYC Health+Hospitals/Gotham Health and Sydenham, New York City, NY USA; 4 Duke Margolis Center for Health Policy, Duke University School of Medicine, Durham, NC USA; 5 Dartmouth Geisel School of Medicine, Hanover, NH USA; 6 Stanford School of Medicine, Palo Alto, CA USA

**Keywords:** Well-child care, Early childhood, Community health workers

## Abstract

This statement from the Pediatric Academic Societies Maternal Child Health: First 1,000 Days Special Interest Group provides an overview of the rationale, evidence, and key action steps needed to engage Community Health Workers (CHWs) into team-based well-child care (WCC) for families in low-income communities. CHWs have been defined as public health workers who have a trusted and valued connection to a community. Integrating CHWs into early childhood WCC can allow for greater cultural relevancy for families, reduce the burden on clinicians to provide the wide range of WCC services, many of which do not require the expertise of a high-level clinician, and improve preventive care services to families during the vulnerable but critical period of early childhood. There are evidence-based approaches to integrating CHWs into early childhood WCC, as well as payment models that can support them. Implementation and spread of these models will require collaboration and engagement across health systems, clinics, payors, and CHWs; flexibility for local adaptation of these models to meet the needs of clinics, practices, CHWs, and communities; publicly available training resources for CHW education; and research findings to guide effective implementation that incorporates parent and caregiver engagement as well as sustainable payment models.

## Well-child care

In the United States, well-child care (WCC) is the only universally recommended and readily accessible program supporting health and development during the critical first 5 years of life. The frequency of visits in infancy and early childhood allows parents and caregivers to develop trusted relationships with a pediatric clinician and their team, build their own knowledge, confidence, and skills to support healthy child growth and development, and gain access to needed community resources to address family and household social and psychosocial needs [1–3]. WCC, however, has been critiqued as too bloated to meaningfully cover all recommended content and support families’ varied needs; that is, there are many more preventive care services (anticipatory guidance, social needs assessment and resource referral, parenting support, behavioral and developmental screening, surveillance, and counseling), than can be provided in a 15-minute visit [4–6]. This mismatch between time available for a visit and the services to be provided is most critical for families living in poverty [7]. Innovative approaches that expand the visit from a single primary care provider to a team approach that engages community health workers (CHWs) in care have evidence of effectiveness in improving child and family outcomes [8, 9]. Yet the literature on CHWs rarely includes discussion of their role in pediatrics and specifically, their potential to support families during the early childhood period as an integral part of WCC. Moreover, team approaches have not been widely implemented in pediatrics, in part because of lack of clear funding mechanisms to support sustainability.

In May 2022, the Pediatric Academic Societies First 1,000 Days Interest Group gathered in Washington, DC, to discuss the need for the integration of CHWs into team-based well-child care during the early childhood period, the evidence to support it, and the urgency for it to happen. This commentary provides an overview of the rationale, evidence, and key action steps needed to engage CHWs into team-based WCC for families in low-income communities.

Families facing poverty and structural racism might benefit the most from team-based care, yet our system of WCC is not structured to address the wide range of social, developmental, and behavioral needs that families face [7]; this is a reality made worse by the COVID-19 pandemic [10]. The pandemic disrupted early childhood programs such as child care and preschool, with estimates of 300,000 fewer children participating [10]. It remains unclear what the developmental impact of this interruption will be on educational and developmental trajectories across the life course. Given the impacts of the COVID-19 pandemic on the health and well-being of children, the time is now to embrace new models for preventive care during early childhood that can reach and support *all* families with young children. Therefore, our goal is to review the evidence for effectiveness of CHWs in WCC, and provide a call to action to integrate CHWs into team-based WCC during early childhood, particularly for economically and racially marginalized families.

### Community health workers

Community health workers (CHWs) function as part of a healthcare team to improve care in marginalized communities that face structural disadvantages, both in the US and globally. CHWs often serve as a bridge between the culture of medicine and the patient, and are characterized as health professionals “with an in-depth understanding of the community culture and language.” [11]. CHWs often live in the community they serve, bringing unique and valuable skills to address health disparities [9]. Their roles include providing culturally appropriate health education, coaching, social support, and direct services, as well as care coordination, case management, and care navigation, among others outlined in The Community Health Worker Core Consensus Project [12, 13]. The term “Community Health Worker” serves as an umbrella term for a wide range of work titles, a few of these are health advocates, patient navigators, health coaches, and in Spanish “promotoras” or “promotores de salud; however, there are many other roles as well [14].

There is rigorous evidence supporting the value of CHWs in improving adult health; studies have documented improvements in chronic disease management, increased cancer screening, improved patient experiences of care, and reduction in health care costs [15]. However, the evidence for CHWs, particularly in early childhood, is not as robust. There are trials that provide evidence on the role of CHWs in pediatric asthma management, [16] comprehensive care coordination for children with chronic diseases, [17, 18] and in pediatric primary care to improve social needs screening and community resource referral [19] There are a few studies that have rigorously evaluated the incorporation of individuals as part of the early childhood well-child care team in CHW roles, with positive intervention effects; these are described below [20–22].

## CHW models in well-child care

From the initial newborn visit through age 5, the American Academy of Pediatrics (AAP) recommends 12 preventive visits, or check-ups, known as “well-child care.” [23]. National guidelines highlight a range of services that should be provided during these visits, including anticipatory guidance, which is age-specific education, guidance, and support on a range of issues parents and the child will encounter (e.g., food introduction, car seat safety, sleep safety, toilet training); screening and community resource referral for family and household social and psychosocial needs (e.g., housing or food insecurity, post-partum depression), and standardized screening and surveillance, for delays and problems in development and behavior [1]. Further, families often need navigation assistance to access resources that can support caregivers in parenting, or to access and utilize early intervention services when behavioral or developmental needs are identified. Finally, prior to preschool or kindergarten entry, parents may not have regular access to any other child health or development professional – WCC visits can often be the only venue where the critical skill and practice of building and nurturing positive relational health with their child is supported.

The incorporation of CHWs as navigators, coaches, or health educators in team-based WCC can support the wide range of services that families living in low-income communities need. While multiple evidence-based, team-based models for early childhood WCC exist, such as Healthy Steps, [20, 24] not all engage CHWs. They do, however, provide evidence for the impact of team based WCC models that support families in the early childhood period. Below, we describe the findings of trials from three WCC models: Healthy Steps, DULCE, and PARENT. In the quasi-randomized and randomized controlled trials (RCT), respectively, of Healthy Steps and DULCE, a licensed professional (Healthy Steps) or a CHW with postgraduate training (DULCE) was integrated into early childhood WCC, and in the PARENT trial cluster RCT, a CHW in the role of a “coach” without a license or formal postgraduate training was integrated into early childhood WCC.

In the Healthy Steps for Young Children model, a licensed professional (not typically a CHW) partners with the primary care clinical team to provide developmental and behavioral services to parents, including screening, assessment, and guidance [24]. Findings from a controlled trial indicate that Healthy Steps families received more anticipatory guidance, were more likely to have had a developmental assessment, and to be up-to-date with visit and immunization schedules [20, 24, 25]. The quasi-randomized controlled trial of Healthy Steps (*n* = 4896) engaged a licensed professional in the role of “developmental specialist”. Current guidance from Healthy Steps suggests that for the developmental specialist, a “master’s degree in psychology, social work, counseling, early childhood education, or related field is highly preferred” and that a “clinically licensed mental health professional is preferred” [26]. However, it is possible for a CHW without a professional license or advanced degree to fulfill this type of role, as demonstrated in a few Healthy Steps sites (personal communication with Zero to Three).

The Developmental Understanding and Legal Collaboration for Everyone (DULCE) intervention is based on the Strengthening Families approach and builds on Healthy Steps and Medical Legal Partnerships [21]. It integrates a CHW (with training in child development or a related field) as a “family specialist” WCC from birth through 6 months of age. The DULCE family specialist meets with families at their infant’s WCC visit, makes home visits, and communicates via email, phone or text, or in-person between these encounters. In the RCT of DULCE with 330 families, the family specialist spent a median of 1.1 h with each family during a WCC visit. Researchers found intervention families were more likely than control to have received their third DTaP vaccine on time (i.e., by 7 months of age, 78% vs. 63%, *p* = 0.002), less likely to have had an ED visit by 6 months of age (37% vs. 49%, *p* < 0.03), and more likely to have completed 5 or more WCC visits by age 1 year of age. Intervention families also received more social needs-related resources (i.e., food, utilities) compared with control. The intervention was from birth through 6 months of age, and at the 12-month follow-up, findings in ED use and immunization receipt still favored the intervention group, but no longer reached statistical significance [21].

Despite this evidence of team-based care for early childhood WCC, the Healthy Steps Specialist in trials has not been a CHW, and thus does not provide direct evidence of CHWs in early childhood preventive care. DULCE does provide this evidence, however, as it engages a CHW as a Family Specialist. Parent-focused Redesign for Encounters, Newborns to Toddlers (PARENT) [27] is a community-designed WCC delivery model that integrates a CHW as a PARENT coach as part of the WCC team for all children ages 0–3, to provide anticipatory guidance, psychosocial and social needs screening and resource referral, and developmental/behavioral monitoring. An initial pilot randomized controlled trial of PARENT employed a master’s degree-level health educator as the coach, and findings indicated intervention effects of improved receipt of WCC services, better parent experiences of care, and a 50% reduction in emergency department utilization [28, 29]. A larger cluster RCT of the PARENT intervention in partnership with two federally qualified health center organizations, and 10 of their clinical sites in CA and WA State, [30] enrolled 914 families, and partnered with “PARENT Coaches” who had either high school or college as their highest level of education, and had previously worked in the role of care coordinator, family advocate, or medical interpreter. Findings indicated that intervention families received more well-child care services (anticipatory guidance, psychosocial assessment, and developmental and behavioral needs addressed), and were more likely to be up to date on well-child care visits [22]. There was no difference in ED utilization, but since the trial occurred over the pandemic, ED rates for both intervention and control were lower than expected. In a post hoc analysis of families who were enrolled just prior to the pandemic, the intervention buffered the pandemic-related decline in maternal mental health outcomes observed among control participants [30].

In the interventions described above, CHWs, or individuals in CHW roles, positively impact families by providing information using their technical knowledge and skills (i.e., safe sleep guidance), and by building supportive and trusting relationships with families. Future studies can help understand the relative contribution of each to overall intervention impact.

Although the focus of this report is on integrating CHWs into the team providing clinic-based, early childhood support for low-income families, it is important to recognize that community-based CHW models for early childhood have been implemented in various non-clinical settings. These models employ CHWs at the level of health plans (e.g., integration of CHWs into state Medicaid plans via Medicaid 1115 waivers), [31, 32] community-based organizations, [33] public health departments, [15] schools, and community-based organizations. Data from trials evaluating these programs within early childhood WCC is lacking, but evidence of effectiveness exists from programs focused on CHW-based asthma interventions [34, 35].

### CHW implementation

There are multiple barriers and facilitating factors to implementing CHW interventions in primary care settings for early childhood WCC, including determining appropriate ratios, certification, and ongoing training [36]. Clinics must first determine for whom and what the CHW support is most needed, and can be most efficiently engaged. CHWs’ role in early childhood can span comprehensive WCC services, focus on developmental needs during early childhood, or focus more on social needs identification and resources. A focus on children ages 0–5 will require training in developmental and social-emotional needs of young children, navigation of early childhood systems, and knowledge of Bright Futures guidelines for preventive care. CHW staffing requirements will vary based on the types of interventions provided, for example, one model estimates 1 CHW is needed for every 176 children with special healthcare needs [37]. Models that focus on system navigation alone may have higher ratios, [14, 38]. while models that are relationship-based and provide more comprehensive WCC services may tolerate lower ratios.

Robust training and ongoing professional development are critical. Certification programs may help ensure candidates have foundational knowledge and skills, and in some places, are a requirement for billing. CHW certification requirements may be a barrier to scaling programs, increase costs, and may not provide the knowledge and skills required for early childhood-specific roles, such as child development, family dynamics, or the impact of trauma on child health. Clinics serving young children would benefit from publicly available training materials in these components of early childhood preventive care. For example, the PARENT training curriculum and implementation guide are available without a cost or registration fee [39]. Another model involves community-based organizations (CBOs) partnering with primary care settings to provide tailored training. Furthermore, the CBO can employ CHWs to be placed in healthcare settings [40, 41].

### State of the field: financing community health workers

There is a wide range of publicly financed mechanisms by which states are engaging with CHWs for both pediatric and adult populations. We (EA/JL) conducted a landscape analysis of pediatric reimbursement models for CHW services of 50 states and 1 District as of July 10, 2023. Our methods included a standardized online search followed by review by the authors and select external child health experts of a subset of states which were all verified as accurate. We were able to identify 18 states that reimbursed CHWs directly through Medicaid and 10 more where MCO’s or ACO’s reimbursed or directly hired CHWs. Overall, 28 states and 1 District had publicly financed CHW programs for children, while 25 states had programs for adults.

### Payment models and metrics

CHW models for early childhood struggle with the challenge of demonstrating a short-term financial return on investment (ROI) for interventions focused primarily on prevention [42]. Most adult CHW reimbursement models focus on high-risk, chronically ill patients, and utilize traditional payment arrangements that define value as short-term savings [43]. Longer-term health outcomes, cross-sector involvement, and family-level cost savings may be needed to understand true cost effectiveness of CHW interventions that can promote high-quality primary care in early childhood [44]. Alternative payment models (APMs) may allow us to re-focus on longer-term outcomes and ability to address patient and caregivers’ needs across sectors. This includes leveraging Medicaid opportunities such as 1115 Waivers, pilots similar to “in-lieu of services” or Patient-Centered Medical Home/Center of Excellence, and state plan amendments [45–47].

There are two broad categories of payment models for CHWs in early childhood WCC – fee-for-service and population-based payment. In recognition of a need to support high-quality, more equitable primary care [42]; some states have adopted Medicaid primary care population-based payment (PBP) models where payment is linked to quality incentives [48]. Because outcomes of interest for early childhood WCC models of care are long term, cross sector, and family-level, process measures with evidence as facilitators of long term improvements in health may need to be used as quality incentives for PBP models (see below for examples).

### Fee-for service payment

Often multiple and complex requirements serve as barriers to successful implementation in fee-for-service systems [49]. Some of these include requirements of a specific diagnosis code to bill, which often limits the population served to those with chronic illness or established risk (e.g., homelessness), CHW credentialing, and the reality that the fee-for-service payment does not meet the clinic’s costs of delivering the CHW provided service. Tiered rates based on risk can be considered but must include adequate payment to support the large number of patients who require less intensive support and must recognize that escalating care needs are not always predictable, especially in early childhood. Tiered rates that include maternal and family risk factors (e.g., teen parent or maternal mental health condition) may also be needed. It is important to recognize children with medical complexity, an important but small proportion of patients in primary care, require more complex care coordination, navigation, and support that would require additional considerations [50].

### Population- based payment

An APM can utilize a per member per month or an additional payment added to each WCC visit to support CHW integration in early childhood WCC. An eligible population would be defined as using primary care services, Medicaid or CHIP eligible, and child aged 0–5 years; practices would need to meet eligibility criteria (e.g., employing an eligible CHW and defining scope of CHW-early childhood care). For adult and pediatric health systems with a total cost of care global arrangement, an explicit requirement for savings to go to pediatrics is needed, given the inherent risk that any system surplus could shift to cover adult care.

### Pediatric CHW metrics

Early childhood CHW outcomes could include both process and outcome metrics. The CHW Common Indicators can serve as a foundation of standard measures to assess CHW practice in early childhood WCC [51, 52]; however additional child health-focused measures will be needed as well. Process outcomes may include the provision of anticipatory guidance, or completion of developmental screening, social-emotional screening, or health related social needs (HRSN) screening. Outcome metrics might include rates of CHW-facilitated referrals or closed-loop completed referrals to early intervention, child care, pre-kindergarten, parenting programs, WIC, or to HRSN resources such as food banks. Health care utilization metrics such as well-child care attendance or decreased emergency department use could be measured [52]. Emerging cross-sector metrics might include rates of school readiness and earlier diagnosis of autism [53]. Notably, all measures may require enhanced data monitoring and tracking systems, including data on patient race, ethnicity, and socio-economic status in order to stratify outcomes. Finally, careful tracking of CHW program elements including costs of hiring, training, and caseloads are needed [52].

### Recommended actions

To promote implementation and scale of CHW interventions in early childhood WCC, we suggest the following considerations for clinical, research, and child health policy and payer communities:

***Create a centralized source for publicly available training resources for CHW in early childhood well-child care.*** Most CHW curricula in primary care are adult-focused. There is a need to make training resources widely publicly available for CHWs whose focus is with pediatric populations. This would allow clinics and practices to implement the core evidence-based elements, while adapting the model or intervention to their own community and needs. This centralized source of training resources would ideally be housed within a CHW clearinghouse, such as the *National Association of CHWs (NACHW’s) Document Resource Center*, making it accessible to the broader CHW community.

#### Establish a national collaborative or working group on CHW in early childhood primary care

As part of the Pediatric Academic Societies’ Maternal Child Health: First 1,000 Days Special Interest Group, the authors of this manuscript attended a session at the 2023 Pediatric Academic Societies Meeting to discuss payment, implementation, and dissemination of early childhood programs. This manuscript is the outcome of that meeting. By organizing as a national collaborative that incorporates CHWs, parents, state Medicaid officers, clinicians, researchers, and policy makers, we can share best practices across states, with the goal of accelerating the uptake and sustainability for incorporating CHWs during early childhood WCC. Such a collaborative should be fully integrated into National CHW organizations, such as NACHW. Current literature and advocacy efforts focus almost entirely on adult-focused models of CHW care. The importance of family level intervention and of a developmental perspective in service delivery, the foundation of pediatric practice, need to be explicitly included in CHW programs and in efforts to build evidence for CHW effectiveness.

***Continue to build the evidence during implementation and spread.*** In partnership with CHWs, families, healthcare providers, and healthcare organizations, we need to employ practical but rigorous evaluations of early childhood CHW programs to understand the effectiveness of interventions, without over reliance on the traditional RCT, in order to build the evidence base. For example, Nationwide Children’s Primary Care Network is employing community-engaged adaptation and implementation of the PARENT intervention across 14 clinics of its primary care network using a stepped wedge clinical trial that allows it to implement the intervention gradually across clinics without the need to randomize patients, or to designate clinics as the control condition [54]. Measuring outcomes using data collected from the electronic health record, health plan administrative data, or even outcome or process measures from other sectors involved in early childhood (e.g., early intervention, WIC, preschool or kindergarten), can further reduce the burden of data collection in traditional trials, further expanding the evidence base.

#### Promote local adaptation of CHW models with parent and caregiver engagement

There is likely not a “one-size-fits all” formula for integrating CHWs into early childhood WCC delivery; local adaptation and implementation of evidence-based CHW interventions will require engagement of the clinical organization’s providers, CHWs, other staff, and leadership, community organizations, and the parents they all serve.

## Conclusions

Released in 2022, the National Academies of Science, Engineering, and Medicine’s Consensus Report, *Implementing High-Quality Primary Care*, defined high-quality primary care as delivered by interprofessional teams that include individuals, such as CHWs [55, 56]. Team-based care that integrates CHWs into the early childhood WCC team can help ensure families receive care that values the lived, culturally-relevant experiences and expertise of individuals who can provide relationship-based preventive care services to families. Integrating CHWs into early childhood WCC can allow for greater cultural relevancy for families, reduce the burden on clinicians to provide the wide range of WCC services, many of which do not require the expertise of a high-level clinician, and improve preventive care services to families during the vulnerable but critical period of early childhood. There are evidence-based approaches to integrate CHWs into early childhood WCC, as well as payment models that can support them; the time is now for the implementation and spread of these models, which will require collaboration and engagement across health systems, clinics, and payors. Given the potential for long term health impacts, national efforts to evaluate and advocate for CHW practice need to include an explicit focus on early childhood CHW practice.

## Data Availability

No datasets were generated or analysed during the current study.

## Abbreviations

WCC: Well-child care
CHW: Community health workers
APMs: Alternative payment models
